# A Comparative Study of Mast Cells Count in Different Histological Grades of Oral Squamous Cell Carcinoma by Using Toluidine Blue Stain

**DOI:** 10.7759/cureus.10626

**Published:** 2020-09-24

**Authors:** Keerthi V Narayan, Grace Sonia, Parikshya Shrestha, Girish Hemadala

**Affiliations:** 1 Department of Oral and Maxillofacial Pathology, Dr. M.G.R. Educational and Research Institute, Chennai, IND; 2 Department of Oral and Maxillofacial Pathology, Rajarajeswari Dental College and Hospital, Bangalore, IND; 3 Department of Oral and Maxillofacial Pathology, KIST Medical College and Teaching Hospital, Lalitpur, NPL

**Keywords:** research in angiogenesis, oncopathology, mast cell, tumor progression, squamous cell carcinoma, toluidine blue

## Abstract

Background

Oral squamous cell carcinoma (OSCC) is the sixth most common cancer worldwide accounting for 90% of all malignant oral lesions with high mortality and a five-year survival rate of about 50%. Various studies have shown mast cells regulate carcinogenesis by immunosuppression, angiogenesis enhancement, and promotion of tumor cell mitosis.

Aim

Hence, the present study was aimed to compare mast cell counts in normal oral mucosa with histological grades of oral squamous cell carcinoma by using toluidine blue stain.

Methodology

Sixty formalin-fixed, paraffin-embedded tissue samples included 15 well-differentiated, 15 moderately differentiated, and 15 poorly differentiated OSCC, as well as 15 cases of the normal oral mucosa (control), were sectioned and stained with 1% toluidine blue.

Results

We observed that the mean mast cell (MMC) count was comparatively more in normal mucosa than in various grades of OSCC. It was higher in low-grade OSCC. However, the differences between grades were not statistically significant.

Conclusion

In the present study, according to the results obtained, the MMC count was significantly decreased in OSCC in comparison with normal oral mucosa. Therefore, it can be assumed that mast cells could serve as an indicator of tumor progression.

## Introduction

Oral squamous cell carcinoma (OSCC) represents 90% of head and neck malignant tumors. OSCC can be broadly described as a malignant neoplasm of stratified squamous epithelial origin with the highest recurrent invasion. Alcohol consumption and tobacco smoking still remain the primary etiological agents of OSCC, along with predisposing factors such as ultraviolet radiation or human papillomavirus infection. Histologically, SCC of the head and neck region is categorized as a well-differentiated, moderately differentiated, or poorly differentiated type based on the nature of the cell differentiation [1].

Mast cells (MCs) are large connective tissue cells with numerous basophilic cytoplasmic granules rich in histamine and heparin, which originated from hematopoietic bone marrow precursor cells and are frequently found scattered along the capillaries. In 1877, Paul Ehrlich identified the MC and termed it as mastzellan, which denotes “a well-fed cell” with intracellular granules contained phagocytosed material or nutrients that could be food for neighbor cells [2]. Histologically, MCs are heterogeneous in shape, varying from round, oval, or spindle-shaped, of about 12 microns (diameter) with 50 - 100 packed granules. It is often difficult to distinguish fibroblasts from MCs in routine hematoxylin and eosin (H&E) stain owing to its similar staining characteristics. Hence, a selective metachromatic stain of 1% toluidine blue was used first in 1856 by British chemist William Henry Perkin who illustrated purplish-red MCs with the nuclei appearing sky blue in color [3].

Numerous studies have shown the association of these normal cells with resistance and susceptibility in the tumor advancement process. Because of these unique properties, these cells are ideally poised to aid as 'gatekeepers' of the normal and tumor microvasculature in the oral cavity [4-5]. Angiogenesis is the growth of new blood vessels from the preexisting ones that necessitate the continued growth and survival of neoplasms. In many human malignancies, angiogenesis has been regulated by a balance between stimulators and inhibitors of angiogenic factors. It has been hypothesized that the angiogenic phenotype may result from the production of various growth factors, such as fibroblast growth factor-2 (FGF-2) and vascular endothelial growth factor (VEGF) by the tumor cells and/or the down-regula­tion of negative modulators, like thrombospondin-1 (TSP-1) in tissues with dormant vasculature [6-7] However, very few studies were performed over the years to understand the nature of the MC in the tumor angiogenesis and progression. Hence, the present study was aimed to compare mast cell counts (MMCs) in normal oral mucosa with various histological grades of oral squamous cell carcinoma by using toluidine blue stain.

## Materials and methods

The study was conducted on 60 formalin-fixed, paraffin-embedded tissue samples that were retrieved from the archives of the Department of Oral and Maxillofacial Pathology, RajaRajeswari Dental College and Hospital, Bangalore. The study included 15 cases of histologically confirmed, well-differentiated OSCC, 15 cases of moderately differentiated OSCC, 15 cases of poorly differentiated OSCC, and 15 cases of the normal oral mucosa. The paraffin infiltrated tissues embedded in wax blocks were sectioned to a thickness of 5 μm using a soft tissue semi-automatic microtome. One set of the section was subjected to routine H&E staining to re-confirm the histopathological diagnosis. Another set of the section was stained by acidified 1% toluidine blue as per protocol for MCs metachromatic staining technique. All stained sections were examined under a binocular light microscope. Five areas were randomly selected at low power (4x) to identify the area of hot spots and the MCC was evaluated under high power (40x) for each specimen. The areas under one high-power field (HPF) were taken as one microscopic field (photomicrographs 1, 2, 3, 4).

**Figure 1:**
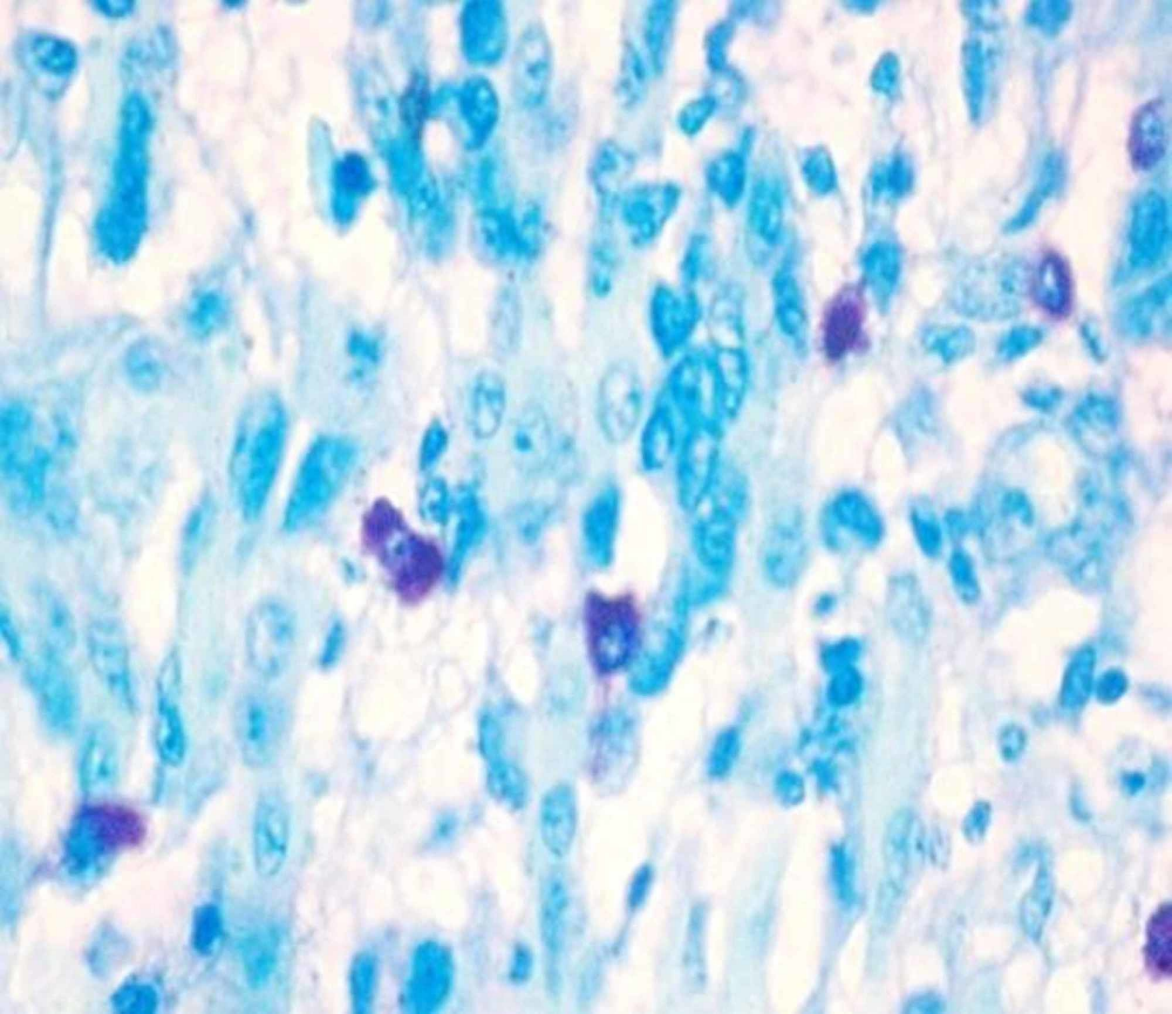
Photomicrograph 1: toluidine blue stain showing mast cells in well-differentiated oral squamous cell carcinoma under high power (40x)

**Figure 2:**
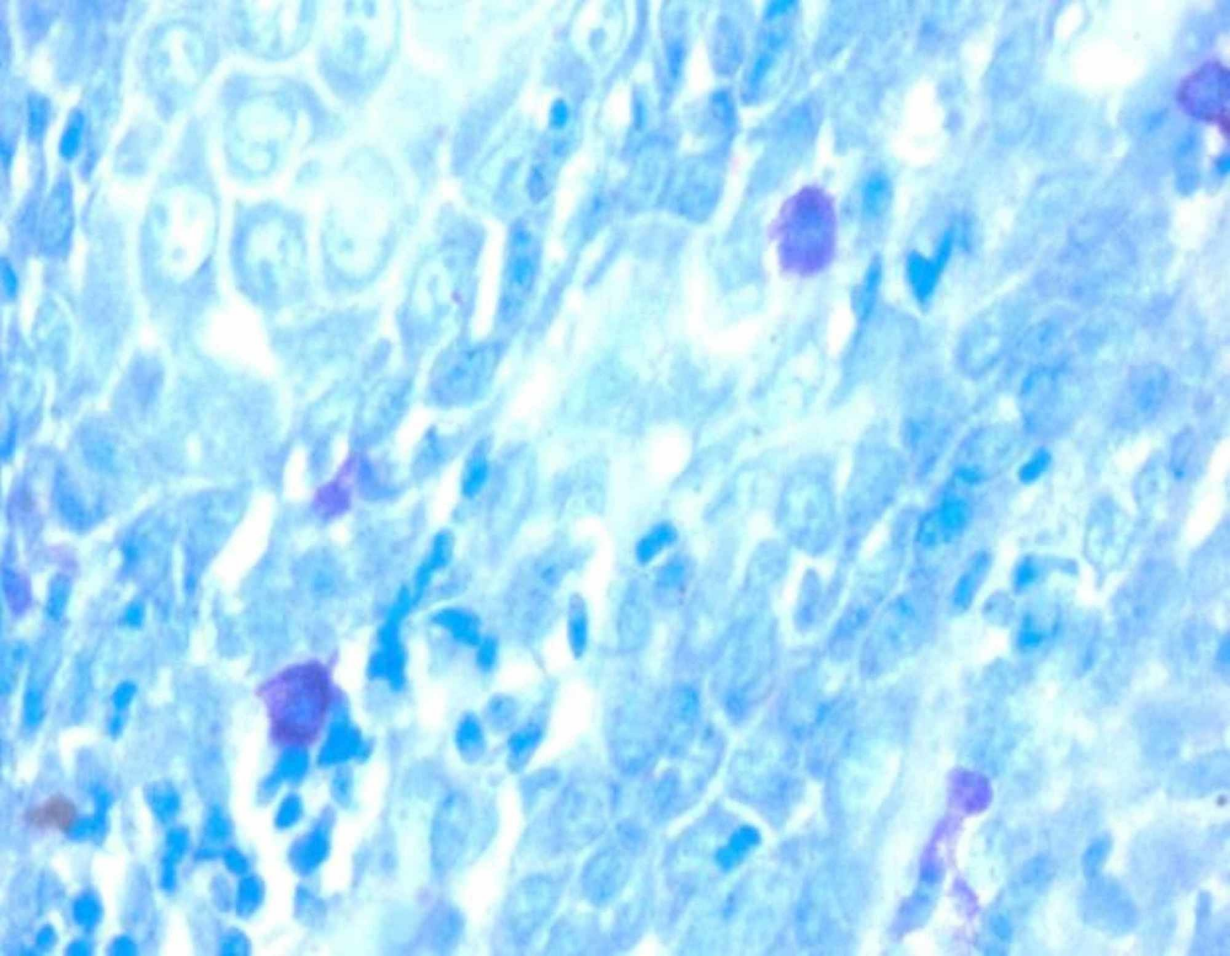
Photomicrograph 2: toluidine blue stain showing mast cells in moderately differentiated oral squamous cell carcinoma under high power (40x)

**Figure 3:**
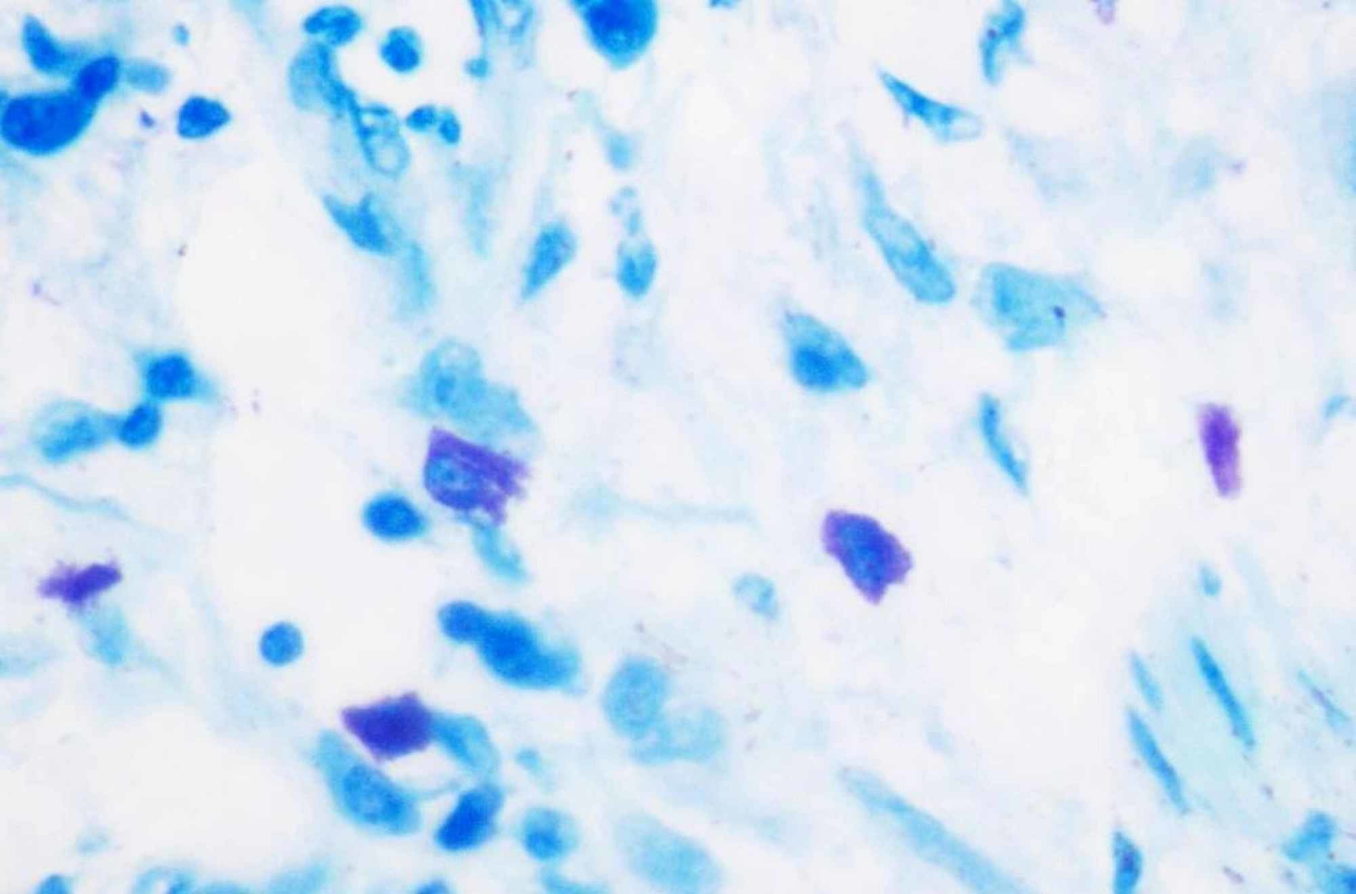
Photomicrograph 3: toluidine blue stain showing mast cells in poorly differentiated oral squamous cell carcinoma under high power (40x)

**Figure 4:**
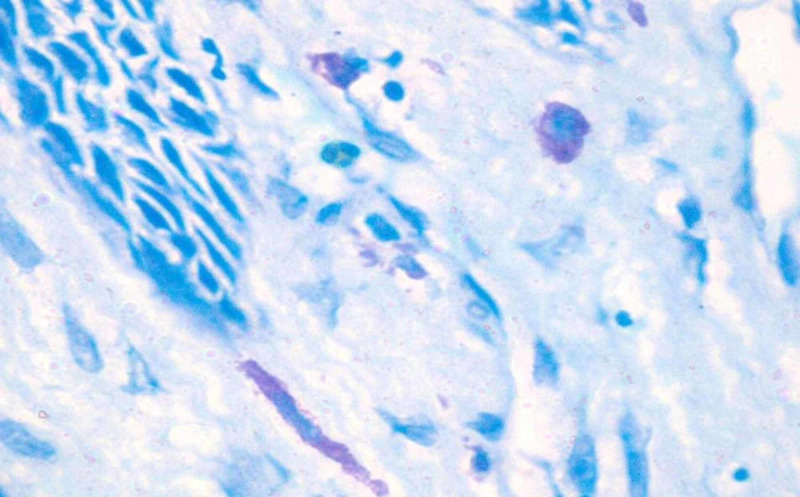
Photomicrograph 4: toluidine blue stain showing mast cells in normal oral mucosa under high power (40x)

Evaluation of mast cells and analysis

Under the light microscope, MCs appearing as pinkish/purple-colored spindle to oval-shaped cells with sky blue-colored nuclei were evaluated. Counting was carried out in five random high-power fields (40x) having a larger number of MCs, with one field depth from the basement membrane of the epithelium and the average per high field (HF) were determined, further the MCC was expressed per square mm within the radius of one field under the high-power objective (40x). All the data were tabulated and a one-way analysis of variance (ANOVA) test was performed to compare and evaluate the number of MCCs in histologically diagnosed OSCC and normal mucosa. This was done to compare and evaluate the MCC in well-differentiated OSCC, moderately differentiated OSCC, and poorly differentiated OSCC using the Statistical Package for Social Sciences (SPSS), version 19.0 (IBM SPSS Statistics, Armonk, NY).

## Results

The study comprised 60 samples including 15 cases of well-differentiated OSCC, 15 cases of moderately differentiated OSCC, 15 cases of poorly differentiated OSCC, and 15 cases of normal mucosa. One-way ANOVA performed to compare the mean MCC between the three grades of OSCC (well-differentiated, moderately differentiated, and poorly differentiated) and the control group (normal mucosa) showed a p-value of 0.1311. The p-value was not found to be significant at α = 0.05 (p < 0.05), denoting there was no substantial difference in the mean MCC among the study groups (Table 1). The paired t-test was performed for comparisons within the groups which showed the mean MMC was comparatively more in normal mucosa than in various grades of OSCC, and it was higher in low-grade OSCC in comparison with well- and moderately differentiated OSCC (Table 2).

**Table 1:** The Mean Mast Cell Count and Observed P-Value by One-Way ANOVA *p-value is not significant (P < 0.05 significance level) ANOVA: analysis of variance; OSCC: oral squamous cell carcinoma

STUDY GROUPS	Mean Mast Cell Count	p-value (One-way ANOVA)
Normal mucosa (Control )	7.7	0.131*
Well-differentiated OSCC (WDSCC)	7.1	
Moderately differentiated OSCC (MDSCC)	4.7	
Poorly differentiated OSCC (PDSCC)	4.5	

**Table 2:** The Mean Mast Cell Count and Observed P-Value Within the Groups by T-Test MDOSCC: moderately differentiated oral squamous cell carcinoma; PDOSCC: poorly differentiated oral squamous cell carcinoma; WDOSCC: well-differentiated oral squamous cell carcinoma

COMPARISON GROUPS	t-value	p-value
Normal mucosa vs WDSCC	-0.081741	0.9355
Normal mucosa vs WDSCC	-1.6334	0.1136
Normal mucosa vs WDSCC	-1.6709	0.1067
PDSCC vs MDSCC	-0.12663	0.9002
PDSCC vs WDSCC	1.8326	0.07752
MDSCC vs WDSCC	1.7491	0.09165

## Discussion

Oral cancer is often diagnosed at the later stages of the disease where most individuals present with localized or regional involvement of the tumor cells (37% localized, 43% regional site, 10% distant or specific) [8]. In India, OSCC is one of the leading causes of increased mortality rates in head and neck malignancies. Studies have shown prognosis and five-year survival rates range from 79% for those with localized disease, 42% for regional disease, and 19% for distant metastasis cases [7, 9]. Progression of oral cancer proceeds through distinct molecular genetic alterations and mutations that are acquired from the loss of genomic integrity following exposure to associated risk factors over a prolonged period of time [6-7]. Neovascularization or angiogenesis is an essential component in a number of regular physiologic processes, including growth and development, healing of oral wounds, and reproduction. It is also considered a critical step in the growth and progression of tumor cells. MCs are effector cells of inflammatory and allergic response/stimuli originated from multilineage hematopoietic progenitors that migrate to tissues and organs where they eventually mature and reside [10-12]. MCs are a well-recognized source of various pro-inflammatory mediators and express the high-affinity IgE receptor on their cell surface.

Various studies have shown MCs regulate carcinogenesis by immunosuppression, angiogenesis enhancement, and promotion of tumor cell mitosis. Increased accumulation of MCs within tumor environments has been correlated with poor prognosis, increased metastasis, and reduced survival in melanoma, adenocarcinomas, squamous cell carcinoma, Hodgkin lymphoma, and B-cell leukemias [13-14]. Our study results were similar to results obtained in the study done by Dastpak et al. and Cheema et al. where MCs were decreased in OSCC in comparison with the normal oral mucosa, suggesting that MCs do not play an important role in tumor progression [6, 15]. In the present study, the mean MCC between the groups was insignificant, similar to results obtained in the study done by Dastpak et al. where the average amount of MCs decreased in OSCC in comparison with normal oral mucosa [6]. The result of this study is similar to the study by Oliveira-Neto et al. where they found that the MC density was lower in OSCC and in premalignant lesions in comparison with the control group [16]. This could be attributed to the migration failure of the MCs, which possibly is reflected as an adaptation variation in the microenvironment during the tumor initiation and progression. From the present study, it can be observed that mast cells are the key immune cells present in the tumor microenvironment. The receptors present on the surface upon stimulation may release proangiogenesis factors, such as vascular endothelial growth factor (VEGF), basic fibroblastic growth factor (bFGF), transforming growth factors, IL-8, matrix metalloproteinase, tryptase, and chymase that participate in the neoangiogenesis or vascular formation process. Kalra et al., Michailidou et al., and Tahir et al. observed that the MCC in high-grade OSCC was significantly higher than normal oral mucosa, so these studies are in contrast with the present study [7, 17-18]. This difference may be due to the difference between the location of tumors and the demographic parameters of our study, such as the patient’s age and sex.

## Conclusions

In the present study, the highest MCC was seen in normal tissue and it was higher in low-grade OSCC in comparison with high-grade. However, the differences between groups were not statistically significant. The same result was seen between high-grade and low-grade OSCC. Therefore, we conclude that the mean MCC was decreased in OSCC in comparison with normal oral mucosa. Hence, it can be assumed that MCs could serve as a possible indicator of tumor progression. However, further studies on the evaluation of various mediators are prerequisites to draw a conclusion on the exact role of MCs in tumor progression.
